# Incomparability and Incommensurability in Choice: No Common Currency of Value?

**DOI:** 10.1177/17456916231192828

**Published:** 2023-08-29

**Authors:** Lukasz Walasek, Gordon D. A. Brown

**Affiliations:** Department of Psychology, University of Warwick

**Keywords:** incommensurability, incomparability, judgment, decision-making, utility, choice

## Abstract

Models of decision-making typically assume the existence of some common currency of value, such as utility, happiness, or inclusive fitness. This common currency is taken to allow comparison of options and to underpin everyday choice. Here we suggest instead that there is no universal value scale, that incommensurable values pervade everyday choice, and hence that most existing models of decision-making in both economics and psychology are fundamentally limited. We propose that choice objects can be compared only with reference to specific but nonuniversal “covering values.” These covering values may reflect decision-makers’ goals, motivations, or current states. A complete model of choice must accommodate the range of possible covering values. We show that abandoning the common-currency assumption in models of judgment and decision-making necessitates rank-based and “simple heuristics” models that contrast radically with conventional utility-based approaches. We note that if there is no universal value scale, then Arrow’s impossibility theorem places severe bounds on the rationality of individual decision-making and hence that there is a deep link between the incommensurability of value, inconsistencies in human decision-making, and rank-based coding of value. More generally, incommensurability raises the question of whether it will ever be possible to develop single-quantity-maximizing models of decision-making.

Many languages include admonishments against comparing things that cannot, or should not, be compared. In English, apples and oranges allegedly cannot be measured against each other. Other languages make the same point using fruits and vegetables (pears, sweet and nonsweet potatoes, and bananas are typically involved). These allegedly noncomparable items typically do have features in common with one another (e.g., sweetness, calorific content, weight), however, and hence their attributes may be comparable even if the objects cannot be compared as wholes. More extreme examples appear elsewhere. Thus it is plausibly claimed that grandmothers cannot be compared with either machine guns (Romanian) or toads (Serbian), that the height of a tower cannot be compared with the loudness of a thunderclap and that warmth and softness are not comparable (Finnish and Russian, respectively), that love cannot be compared with the eye of an axe (Spanish), and that gingerbread and windmills cannot be compared (Polish) any more than horses and cattle can mate (Chinese).^1^

But if things cannot be compared, how can we choose between them? Whether comparison is possible or impossible can be asked of individual attributes of a single object *i* (height_*i*_ and loudness_*i*_), of individual attributes of different objects (height_*i*_ and height_*j*_ or height_*i*_ and weight_*j*_), or of whole objects (toads and grandmothers). It can be asked about either magnitudes (we can say that grandmothers are larger than toads, although we cannot say that a tower is taller than a thunderclap is loud) or about preferences (we might prefer grandmothers to machine guns or the reverse). Here our concern is with the comparability and commensurability of value in the specific context of preferential choice. Issues of value comparability lie at the heart of models of decision-making and choice, because to say that one item is preferred to another is (at least) to say that it scores more highly on some positively valenced criterion. But what is this criterion, and how universal is it?

The question of what makes it possible for commodities to be valued, compared, exchanged, and priced in a common currency has a long history in economics that both predates and informs current models of choice. Marx, for example, addresses this very problem in the introductory pages of *Das Kapital*. He, like Smith and Ricardo before him, distinguished between *use values* and *exchange values* (Marx, 1867/1976; Ricardo, 1817; Smith, 1776/1937). Marx assumed, as we will also assume here, that use values are not commensurable,^2^ or at least not commensurable in any sense adequate to underpin their exchange value. His theory of value posited instead that commodities are exchangeable at rates that are ultimately rooted in the amount of labor that goes into their production. But labor theories of value, whatever their merits and demerits, do not relate to the problem of how a chooser could compare the use values, for the chooser themselves, of different types of goods. Equally priced options may have different (actual or anticipated) consumption utilities, and these utilities may differ between individuals. One approach is therefore to distinguish between use values (properties of objects) and utilities (subjective or inferred quantities) and to assume that the latter can be compared even if the former cannot (see, e.g., Sinha, 2019). Whether or not this distinction is coherent (for it is difficult to make sense of an object’s use value independently of the utility the object’s possession might confer), Marx did not make use of it as a solution to incommensurability.

A very different approach arrived with the marginal revolution, dating from around the 1870s and associated with Jevons, Walras, and Menger. The labor theory of value was largely abandoned (at least as the foundation of a theory of price determination), and the focus switched to marginal utility as a form of common currency. This utility-based approach to the commensurability problem has survived in various forms until the present day (see Moscati, 2018). However, the move from the labor theory of value to marginal utility did not solve the problem of incommensurability, despite the fact that it has been implicitly assumed to do so both by neoclassical economic approaches and by modern psychological theories of choice. Rather, we suggest, Marx’s and others’ concern with the incommensurability of use values cannot be solved by replacing “use values” with “utilities” and hence remains unaddressed.^3^ Although difficulties with the idea of a single utility have been noted within economics (Georgescu-Roegen, 1954; Sen, 1980), these difficulties are not reflected in recent models of individual decision-making within either economics or psychology.

In this article we discuss incomparability and the related concept of incommensurability as they relate to psychological models of people’s preferences, values, and everyday choices. We suggest that many choices, both those that are met in everyday life and those that are studied by behavioral scientists, are difficult to explain and predict because of the assumption of commensurability (Levi, 1986). Our central suggestion is that, contrary to widespread assumption, there is no general utility-like overall psychological scale with respect to which all choice objects or their attributes can be valued (or can be understood as being valued). Although we argue against the assumption of a universal scale of value, it is difficult to “prove a negative.” Instead, our strategy is to note that a number of otherwise puzzling phenomena become less surprising if we abandon the common scale assumption. More specifically, many phenomena are difficult to reconcile with the universal-value-based approach to decision-making but are natural if we assume value incommensurability.

Our first claim is therefore that choices are made using context-specific values, which we call “covering values.” Because our argument is that different covering values are incommensurable, that is, that there is no universal covering value, this claim raises problems for most existing models of choice in psychology, economics, and neuroscience and more generally for any account that attempts to explain people’s choices as maximizing some single quantity. As our second contribution, we discuss the consequences of abandoning the assumption of a universal covering value for models of decision-making. We also discuss what types of decision model could accommodate the lack of common currency of value. Such models include lexicographic (noncompensatory) heuristics, which do not require decision-makers to trade off attribute values to make a choice. Our conclusion is that although some heuristics may help decision-makers to choose, value incommensurability necessitates the use of *rank-based* processes for decision-making. Finally, we note that reliance on rank-based strategies leads inevitably (by Arrow’s impossibility theorem) to inconsistences in decision-making, such as preference reversals, of the type that are typically observed in experimental studies of choice.

## Commensurability and Comparability

Terms such as “incommensurable” and “incomparable” have been used in different ways by different scholars and have sometimes been treated as synonymous. Chang (2013), however, outlines five different ways in which the term “incommensurability” has been used (see also Chang, 2002) and also distinguishes between commensurability and comparability. Here, following Chang and others, we will take claims about commensurability to be claims about values (rather than claims about objects or their attributes, which are merely the bearers of value). Specifically, we will say that two values are incommensurable if there is no higher-level common scale of value (such as utility, happiness, or inclusive fitness) using which they can themselves be compared.

**Fig. 1.**
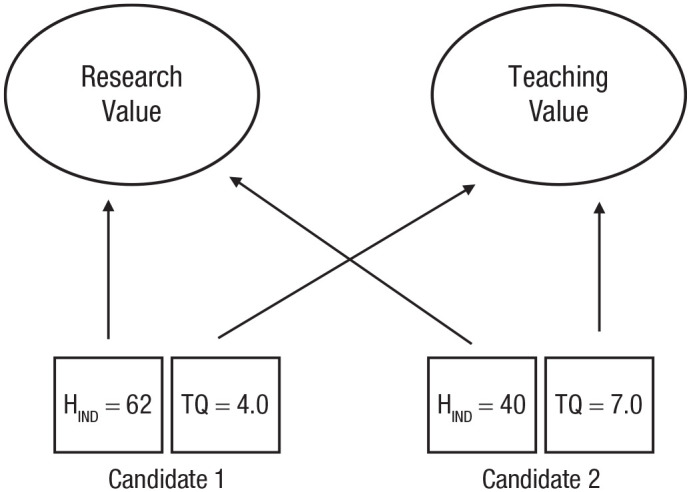
Illustration of a multiattribute choice where there are two covering values (“research value” and “teaching value”) that are not themselves comparable with respect to a higher-level common scale of value. H_IND_ = h-index; TQ = teaching quality (e.g., students’ ratings).

We rely also on the distinct notion of comparability. Comparability (or its absence) is different from commensurability (or its absence) because it is a property of objects or their attributes, not a property of values. Again following Chang, we take comparability as being necessarily *with respect to* some covering value.^4^ We define a covering value as a value that provides a context-specific common currency that enables comparison of attributes of choice objects.^5^ To exemplify: Consider the multiattribute choice (illustrated in Fig. 1) between two candidates for an assistant professor position in a research-intensive university. Assume that there are two different values with respect to which these two candidates could be compared. One is research value, which can be measured using citation counts (e.g., h-indices). The other is teaching value, which we will assume can be measured using student satisfaction ratings. In Chang’s terms, research value and teaching value are different covering values. If only one covering value is relevant to the decision, the choice is easy: Choose the best teacher, or choose the best researcher. The two candidates are comparable with respect to their value as a teacher, and they are comparable with respect to their value as a researcher. The difficulty comes when university guidance on appointments says that teaching and research should both be considered. Research value and teaching value are, we suggest, incommensurable; there is no higher-level, utility-like, abstract common currency such as “academic quality.” We cannot, therefore, say that the candidates are comparable without adding “with respect to *X*” where *X* is a covering value. Because research value and teaching value are incommensurable, selectors must pick one of these two covering values as the basis for decision if they consider the pair of candidates in isolation.

The incommensurability of covering values has implications for whether trade-offs between scores on different attributes are meaningful or not. Trade-offs between any two attribute scores will not be possible if each attribute is relevant to just one, different, covering value. To be concrete: Suppose that candidate A has an h-index of 62 and an average teaching evaluation of 4/10, whereas candidate B has an h-index of 40 but a teaching rating of 7/10. The two h-indices can of course be compared meaningfully with one another, and the two teaching ratings can also be compared meaningfully with one another. However, if teaching ratings indicate only teaching value, and h-indices indicate only research value, there is no way to trade off the two different scores against one another. The h-index difference of 22 cannot be said to outweigh (or not to outweigh) the difference of 3 in teaching rating—because there is no common covering value to enable the trade-off to be made.

However, trade-offs between scores on different attributes can be meaningful just if the attributes contribute to the same covering value. For example, suppose that high extraversion and high conscientiousness both contribute to teaching value. A large advantage on extraversion could then compensate for a small disadvantage in conscientiousness (if the choice between candidates is being made on the basis of teaching value). Thus whether or not scores on one attribute can be traded off against scores on another attribute depends on whether or not the attributes relate to a single covering value. This fact places limits on the ability of noncompensatory models (e.g., “simple heuristics that make us smart”; Gigerenzer et al., 2000) to address the challenge posed by incommensurability in everyday choice. We discuss this issue in more detail later, in the section Rank, Incommensurability, and Heuristics, where we consider how models of choice could respond to incommensurability.

Finally, although commensurability is a feature of values, not of objects or attributes, it is nonetheless natural to speak of the commensurability or otherwise of attributes and attribute values. In doing so, it must be kept in mind that attributes inherit their commensurability or incommensurability solely from the covering values that they relate to.

In arguing that choices cannot be understood in terms of a single universal covering value—that is, in arguing for incommensurability—we focus on everyday multiattribute choices like the one illustrated in Figure 1. We do not address the wider (and much discussed) issue of whether goods such as clean air, loving relationships, human dignity and freedom, or body parts can be compared with each other or assigned meaningful monetary values; neither do we discuss the idea of a hierarchy of values (Scheler, 2017). It is widely assumed in the legal and philosophical literature that violations of rights and principles of justice cannot be compensated by money (e.g., Chang, 1998; Okun, 1975; Sen, 2010). People intuit that some things should not be purchasable and that some trade-offs are taboo (Leuker et al., 2021; Roth, 2007; Sandel, 2012) and that social goods, such as money, health, and education, cannot be rank ordered with respect to one another (Walzer, 1983). The distinction between pluralistic and monist theories of intrinsic value has received much attention within philosophy (e.g., Heathwood, 2015; Scheler, 1973; Smart & Williams, 1973). Our interest, instead, is incommensurability in the more prosaic multiattribute choices that confront individuals in their daily lives, such as between cameras varying in price and memory capacity, apartments varying on rent and location, or jobs varying in pay, location, and hours (see also Levi, 1986). In focusing on the types of choices that people face in their daily lives, we exclude from our consideration choices between risky gambles or lotteries. Although tasks involving such alternatives have been extensively studied in psychology and economics, it has been argued that they are not representative of everyday life choices (Shiffrin, 2022).

## The Ubiquitous Commensurability Assumption

How universal is the assumption of commensurability in decision science? Here we show that the idea of a single, universal covering value, with respect to which choice objects can be preferred, lies at the core of current psychological, economic, and neuroscientific theorizing.

### Economics

The concept of utility, fundamental to most economic theorizing, grew out of the earlier idea that there was a single dimension of pleasure (“happiness”) and that the consequences of actions could be measured in terms of their effects on pleasure or pain (as in Edgeworth’s “hedonimetry”; Edgeworth, 1881, explicitly assumed that all pleasures are commensurable even across individuals). Although Mill acknowledged the existence of different types of pleasure, and the greater worthiness of some types of pleasure than others (poetry vs. pushpin), the basic idea that people could be understood as ideally maximizing a single quantity, happiness, was nonetheless central to utilitarian theorizing (Sen, 1980). Concerns about the subjectivity and measurability of “happiness” led to the more conservative assumption that people’s preferences are revealed by their choices between bundles of products and the replacement in theories of subjective mental states (happiness or well-being) by the notion of utility (an inferred theoretical construct; Bruni & Sugden, 2007; Moscati, 2018; Read, 2007). The approach involves inferring a utility function that systematizes people’s choices. In economic models that presuppose people choose “as if” they were maximizing utility, there is no need to think of utility as reflecting some measurable mental state (Friedman, 1953). Rather, utility is merely a “mathematically convenient way of *describing* the individual’s choice” (Craswell, 1998, p. 1424, emphasis in original). Of course, such an approach is possible only to the extent that people’s choices are consistent. This “as-if” interpretation of utility is not, however, shared by economic models that incorporate psychological processes and constructs.

Finally, in the growing field of cognitive economics, it is now common to assume that decision-makers derive utility from the content and consistency of their beliefs (e.g., Brown & Walasek, 2020; Hertwig & Engel, 2016; Molnar & Loewenstein, 2021). However, such models continue to use the concept of utility as a common currency (Piantadosi & Hayden, 2015a).

In summary, even recent economic models of choice inherit from older happiness-based approaches the assumption of commensurability (i.e., comparability of choice objects with respect to a single construct, utility). This assumption is represented by a preference-completeness axiom or by restrictions of analysis to cases where choices are actually made.

Some economic models allow for incomplete preferences (see, e.g., Dubra et al., 2004); such models typically assume that, absent commensurability, little can be said about choice.

### Models of judgment and decision-making

Traditional psychological models of choice, although emphasizing cognitive mechanisms, normally also assume a single and universal scale of value. For example, prospect theory—the dominant descriptive model of choice—is framed in terms of “value” rather than utility, emphasizing the psychological nature of the model, but nevertheless assumes value commensurability. Vlaev et al. (2011) identify three classes of psychological choice models. Type 1 models, such as TAX (Birnbaum, 2008), prospect theory (Kahneman & Tversky, 1979), and multiattribute utility theory (Keeney & Raiffa, 1993), are “value first” and assume a common scale of value. Type 2 models, or comparison-based models with value computation, allow for comparison-related context effects at the level of both attributes and whole objects. Models in this category nevertheless assume that any choice option can be assigned a value on a single universal scale. A third type of model identified by Vlaev and colleagues assumes that decision-making occurs without value computation in the normal sense. A related class of models has been developed in the “simple heuristics that make us smart” tradition (see also Lavoie, 2014). We return to this third class of model later, as such models can be seen as responses to concerns about comparability and commensurability (Artinger et al., 2022).

The intended interpretation of “value” is often not specified in psychological models of choice, but it is occasionally equated with emotional experience (Kahneman, 2000; Mellers, 2000) or feeling (Goel, 2022). A common idea in the decision-making literature is that people “choose what they like” (Zajonc, 1980), although the exact meaning of “liking” varies between models.^6^ In decision affect theory (Mellers et al., 1997), for example, choices between lotteries are assumed to maximize expected emotional response. A simple version of the model is Savage’s minimax criterion, where payoffs are transformed into regret values (Savage, 1951). Regret values represent subjective emotional experiences that serve as a common currency. Many models in judgment and decision-making (JDM) thus assume explicit and common psychological dimensions. This amounts to an assumption of value commensurability. However, there are many different types of affect just as there are many types of happiness.

Many recent developments in JDM focus on specifying the cognitive processes underlying value-based choice. This trend is reflected in the popularity of sequential sampling models (Busemeyer et al., 2019; Clithero, 2018) as well as by new research linking specific process-level data (e.g., eye movements, search behavior) to parametric estimates of value-based cognitive models of choice (e.g., Mormann & Russo, 2021; Pachur et al., 2018). A common property of these models is that information about choice options is accumulated and integrated by a decision-maker into a single value signal that underpins choices. Thus such models also implicitly assume commensurability of attribute values.

In summary, a large body of mainstream quantitative JDM research makes the implicit assumption that empirical violations of economic theory can be captured by a model in which choices nonetheless reflect maximization of a single common currency of value. Some exceptions do exist, including reason-based choice and constructed-preference approaches, and we discuss these in the Related Approaches section.

### Neuroscience

Much research in neuroeconomics seeks to understand whether values of choice options are reflected in the activation of specific brain areas or neural networks. Several comprehensive reviews of this literature already exist (see Tobias & Sander, 2015), and we therefore focus our discussion on claims about the existence of a common neural currency.

It is now well established that the activation of specific subregions of the brain reflects reward size. In particular, ventromedial prefrontal cortex/orbitofrontal cortex (vmPFC/OFC) and the striatum appear to encode the subjective value of rewards, such as amounts of money (Grabenhorst & Rolls, 2011), pleasantness of olfactory experiences (O’Doherty et al., 2000), gustatory experiences (O’Doherty et al., 2002), attractiveness of faces (O’Doherty et al., 2003), and the beauty of natural scenes (Yue et al., 2007). Thus, the same brain regions appear to encode subjective values even when they originate from different sensory modalities. Further evidence comes from studies in which participants must trade off different types of rewards. For example, Smith et al. (2010) found that the posterior part of the vmPFC/OFC tracks the exchange rate between two distinct rewards: money and attractive faces (as well as food and money; see also Levy & Glimcher, 2011). In summary, the idea of a neural common currency of value is represented in much recent work within neuroeconomics. We return to this evidence in a later section, where we argue that available neuroscience evidence findings can be explained without the assumption of a common currency of value.

## Decision-Making Without Commensurability

We have argued that the assumption of commensurability is prevalent in models of economics, psychology, and neuroeconomics. Much research on decision-making takes these utility- or value-based models and attempts to develop and modify them, while retaining the commensurability assumption, to account for the inconsistencies and context effects that are observed in people’s choices. Here we take the opposite tack, suggesting that many examples of inconsistencies and irrationalities in people’s decision-making disappear if commensurability is no longer assumed.

First, people are largely incapable of providing consistent and context-independent valuations of nonmarket goods, such as health, safety, or personal data. Judgments of crime-appropriate levels of punitive damages, for example, are inconsistent and noisy unless a mapping scale is explicitly provided (Kahneman et al., 1998). People also have difficulty in domains that are a more regular part of their everyday experience. For example, they cannot trade off quantities such as money and pain in any consistent way: Vlaev et al. (2011) show that the amount of money that people pay for pain relief is determined strongly by the amount of money they have available. If there were a decision-relevant universal common currency of value, such as utility or happiness, then it would be trivial for individuals to map and trade off goods, such as health, onto other metrics, such as money amount. People’s difficulty in doing so would then be surprising. However, from the perspective presented here, monetary valuations are actually particularly ill-suited for providing a common metric of exchange. Because money can be exchanged for many objects and services, there are many covering values that might be salient to a decision-maker contemplating how to trade extra income off against their health.

The consequences of retaining the commensurability assumption are particularly relevant when we confront preference reversals (e.g., preferring job candidate A over candidate B in one context but B over A in another). Such inconsistencies are typically taken as problematic, because utility functions cannot be easily constructed to accommodate systematic preference reversals. However, preference reversals are theoretically unproblematic if they simply reflect changes (perhaps primed by experimental manipulations) in the covering values with respect to which choices are being made (cf. Levi, 1986).

Consider for example the three widely studied effects of context in multiattribute choice: the similarity, attraction, and compromise effects (Wollschlaeger & Diederich, 2020). Models of these effects in both psychology and economics typically assume that the addition or removal of items from a choice context in some way changes the weightings or salience given to different attributes in determining the overall valuation of a choice object (Bordalo et al., 2013; Bushong et al., 2021; Koszegi & Szeidl, 2013; but see Bergner et al., 2019). Context-induced changes in attribute weightings are, however, hard to understand if there is a single common currency of value—because the existence of such a currency mandates a fixed trade-off between attribute values. Once we abandon the common-currency assumption, however, context effects become theoretically unproblematic because they can be assumed to reflect changes in which covering values matter for the decision-maker’s mind (Arkes et al., 2016). In other words, differing attribute weights can be assumed to reflect differing covering values rather than the influence of contextual items per se.^7^ More specifically, many preference reversals found in laboratory tasks may reflect the fact that experimental designs often introduce ambiguity about the relevant covering value (i.e., the covering value with respect to which choice objects are meant to be evaluated). To see this, imagine a thought experiment in which the relevant covering value was made clear to participants by the experimenter. For example, suppose that a participant facing a multiattribute choice between two smartphones is informed that battery life is the attribute they should be most concerned with. With the covering value thus specified, we expect that the classic context effects would disappear. This suggests that at least some context effects can be understood in terms of context-induced changes in covering values rather than in terms of changes in relative contributions of different attributes to a single, common-currency valuation. It also suggests that decision-making problems relating to incommensurability may arise relatively infrequently in everyday life, because in everyday life, there is usually no ambiguity which covering value or which goal is relevant. Indeed, if such ambiguity existed, it could be easily exploited through “money pumps.” Yet, there is little evidence that failures of coherence lead to negative effects on people’s wealth, health, or happiness (Arkes et al., 2016).

The study of mental well-being provides additional results that can, we suggest, more naturally be interpreted if the assumption of a universal common currency of value is abandoned. As noted previously, the idea of a universal utility-like currency of value in economic theorizing developed from the idea that pleasure was unidimensional, at least insofar as it motivated choices. However, both intuition and experimental observation suggest that happiness is a multidimensional construct. Nearly two centuries ago, Berkeley suggested it was difficult or impossible “to frame an abstract Idea of Happiness, prescinded from all particular Pleasure, or of Goodness, from every thing that is good” (Berkeley, 1734, p. 120). Modern research confirms the multidimensional nature of well-being. Thus, for example, numerous recent factor-analytic studies typically find people’s self-reported well-being to have at least three distinguishable components, albeit with a common statistical core (Busseri, 2015; Jovanović, 2015; Kapteyn et al., 2015). Moreover, variables such as income predict some components of well-being (life satisfaction) but not others (affect-related happiness) (Kahneman & Deaton, 2010). Benjamin et al. (2017) provide experimental confirmation of the intuition that people may choose options other than the ones that will make them happiest. The existence of different subcomponents of well-being, while consistent with some evolutionary arguments adduced later, does not in itself disprove the idea that choices can be understood as single-value maximizing. Subcomponents of subjective well-being could be epiphenomenal or irrelevant to actual choice. However, under the perspective presented here, different aspects of self-reported well-being may naturally be regarded as relating to different covering values. Choice options may be good for one aspect of our mental well-being but not the other, and a choice can be difficult if these different facets of mental well-being cannot be matched onto some common scale of value (Sen, 2010). Issues of incommensurability also apply to alternative approaches to human welfare, such as those that seek to maximize capabilities (Sen, 2010); capabilities themselves appear to be incommensurable (Sen, 1985).

## Incommensurability of Covering Values

In this section we explore the implications of the view that there is no common currency of value. What are the consequences of incommensurability for models of JDM that are intended to explain the processes underlying multiattribute choice? Without any overarching utility or value, how could any trade-offs take place? In what follows, we expand our discussion of covering values and outline the general architecture of possible alternative models of choice.

### Four cases of multiattribute choice

We return to the notion of covering value illustrated in Figure 1 and distinguish between four different cases of multiattribute choice. The cases differ in the number of covering values that are present and relevant to the choice. We discuss cases where (a) no covering value is available, (b) there is only one covering value, (c) multiple covering values exist but only one matters for choice, and (d) multiple covering values exist and more than one matters for choice.

#### Type I: no covering value available

We take Type I cases to be rare in practice if we interpret the notion of “covering value” broadly enough. Even items like chalk and cheese could still be compared with respect to their value as, for example, paperweights. In any case, Type I cases are theoretically unproblematic in the sense that no value-based choices can be made when there is no covering value. If people are forced to choose in noncomparable cases, they must choose randomly.

#### Type II: one covering value

Type II cases are also theoretically unproblematic. If there is just one covering value with respect to which two choice objects differ, then no difficulty associated with trading off different covering values arises. In practice, it is difficult to imagine any two objects that share only a single covering value. Even in the case of very different objects, such as cheese and chalk, it is possible to generate multiple covering values, such as “projectile capacity” or “value as a gift” (Chang, 1998). In practice, therefore, decision-makers are unlikely to face choices in which only one covering value is at least potentially relevant.

#### Type III: two or more covering values; only one is relevant at the time of choice

Unlike Type II cases, Type III cases are commonplace in everyday choice. However, Type III cases are also unproblematic, being effectively identical to Type II cases in that the decision is made with respect to just one covering value. The only difference is that other covering values might be relevant at a different time or in a different context (leading to rational preference reversals). Consider again our example of two academic job candidates. If research is the only relevant covering value, then a decision-maker can easily make a choice. Yet it is possible, and indeed likely, that there will be situations in which a decision-maker must compare candidates with respect to another covering value, say, teaching. A choice may be easy to make whichever of the two covering values is relevant at the time of choice, but it is not given that a decision-maker would pick the same alternative in both cases.

Changes in covering values over time may reflect changes in an agent’s state.^8^ For example, we are sometimes more hungry than thirsty, and sometimes the reverse is true. There is nothing mysterious about “preference reversals” (e.g., between a thirst-quenching sorbet and a bag of fries) when they reflect changes in state-dependent wants, but the lack of mystery does not mean that hunger satisfaction and thirst satisfaction are commensurable in that their common values inform choice. The same lack of mystery applies, we contend, when other, more permanent covering values are involved.

#### Type IV: two or more covering values; more than one is relevant at the time of choice

Type IV cases are also common in everyday choice. Situations in which there are multiple conflicting covering values pose the biggest problems to existing decision-making models. There are many situations where more than one covering value exists and is relevant at the time of making a choice. For example, competing covering values might be the research value and teaching value of a job candidate, the location convenience and spaciousness of an apartment, or patriotism and pacifism (as in Levi’s [1986] example about the decision whether to fight for one’s country). When multiple covering values are salient, a choice will be difficult due to the incommensurability of these values. Without any higher-order common currency of value, it is impossible to evaluate objects with respect to two or more covering values. Most value- and utility-based models in psychology and economics do not, we suggest, adequately accommodate Type IV cases.

Even when models are fitted to choice data, this fitting uses results from experiments on multiattribute-choice experiments in which covering value is not specified. In such studies, it is typically unclear to the participant which covering value should take priority in making the choice. In the remainder of this section, we discuss what strategies are available to decision-makers if multiple covering values are relevant in multiattribute choice.

### Decision-making with conflicting covering values

Suppose it is true that in many everyday choice situations, decision-makers must cope with the presence of multiple, relevant, and incommensurable covering values. Theoretical models of decision-making will then need to account for these multiple covering values in multiattribute choice. Here we expand on how models of choice would need to be augmented in the light of incommensurability.

**Fig. 2.**
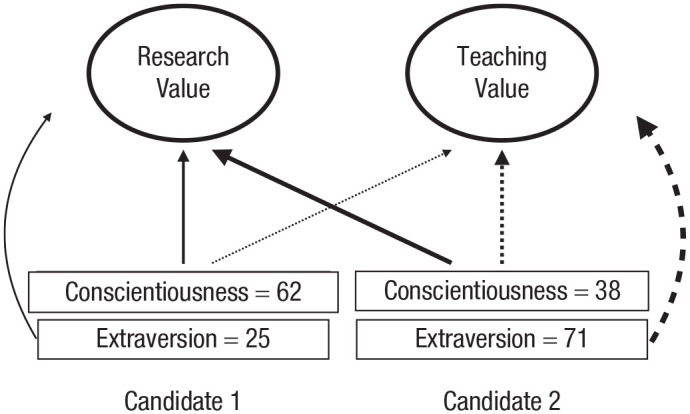
Illustration of a multiattribute choice where there are two incommensurable covering values and different attributes (“extraversion” and “conscientiousness”) can be traded off against one another with respect to each covering value. Not all links are shown.

In contrast to the assumption of most existing models of choice, conflicting covering values necessitate multiple weightings of the same attribute. More specifically, attribute weightings must be covering-value specific. Consider again our example of choosing between two job candidates, where two incommensurable covering values (research value and teaching value) are relevant (Fig. 1). That example is simplified in that each of the two attributes we considered (h-index and teaching-quality rating) is relevant to one and only one covering value. In more realistic cases, a given attribute (and more than one attribute) may be relevant to more than one covering value. We illustrate with a case that involves the same two job candidates and the same two (assumed incommensurable) covering values: one relating to teaching and the other relating to research. However, we now consider the choice relevance of two personality characteristics (conscientiousness and extraversion) as each of these traits is likely relevant, albeit in different degrees, to each of the two covering values as illustrated in Figure 2.

We can then say that the two different attributes, conscientiousness and extraversion, are comparable but, crucially, only with respect to a particular covering value. With respect to the “teaching” covering value, for example, it could be that the weight attached to extraversion is higher than that of conscientiousness. The reverse could be true if the covering value concerns “research.” To be concrete, we could imagine that a 1-standard-deviation increase in extraversion is associated with a 0.7-standard-deviation improvement in teaching ability, while a 1-standard-deviation increase in conscientiousness is associated with only a 0.4-standard-deviation increase in teaching ability.

Thus whereas the different attributes are comparable within the context of a single covering value, they are not comparable across different covering values. In other words, the decision weight attached to extraversion with respect to “teaching value” is not comparable with the decision weight attached to the same attribute established in relation to the “research value” covering value—because the covering values themselves are incommensurable. In Figure 2, the thicknesses of solid lines can be compared because they all relate to a single covering value (research value). However, these solid lines cannot be compared with the dotted lines that link attributes to the other covering value (teaching value).

We can therefore envisage a set of decision weights on attributes that allow choice between different job candidates if the covering value is research reputation. We can also envisage a set of decision weights on attributes that allow choice between different job candidates if the covering value is teaching ability. The decision weights on attributes will, however, be different in these two cases—separate attribute weightings are required for each covering value. Yet virtually all conventional models of multiattribute choice include only a single set of decision weight. Such models will be adequate only when there is only one covering value that is relevant to the decision-maker. If there is more than one covering value, a single set of decision weights cannot suffice.

### Rank, incommensurability, and heuristics

The incommensurability of covering values imposes strong constraints on the types of information that will be useful in decision-making, because information that is incommensurable cannot be combined into a single decision-relevant quantity. The large literature on “simple heuristics that make us smart” (e.g., Gigerenzer et al., 2000) has revealed many cases in which good decisions can be made even if some information is ignored. Here we note the close connection between (a) the incommensurability of values and (b) people’s use of simple heuristics.

The simple-heuristics literature distinguishes between compensatory and noncompensatory decision strategies. According to a compensatory strategy, an option being good in some respects can compensate for it being less good in others (e.g., a car’s poor fuel economy could be overcome by excellent comfort and performance). Under noncompensatory strategies, in contrast, such compensation does not occur (e.g., if fuel economy is less than some threshold value, no amount of comfort or performance can overcome the disadvantage of poor economy).

What, then, is the connection between the incommensurability of covering values and the use of noncompensatory strategies? If different attributes relate to different covering values (as in Fig. 1), then compensation will be impossible because there is no common currency that can be used to effect the compensation. In such cases, a noncompensatory strategy must be used out of necessity, and the question of whether or not it is computationally efficient to do so does not arise. Indeed, when different covering values are involved, there are (in terms of existing approaches) only two basic courses of action available to decision-makers. Either decisions must be made on the basis of just one of the covering values or decisions must be made by counting up the number of covering values on which each option wins (or reach some attribute-specific aspiration level; Artinger et al., 2022). To the extent that there is a one-to-one correspondence between attributes and covering values, many of the simple heuristics that people used in judgment and decision making, such as elimination by aspects (Tversky, 1972), the priority heuristic (Brandstätter et al., 2006), and tallying (Dawes, 1979), can be seen as versions of one or other of these two approaches. Heuristics such as these are consistent with (and may reflect) incommensurability in that they are methods for inference and choice that do not assume that different covering values can be traded off against one another. However, these heuristics *do* often assume that the relevant attributes or covering values can be ordered with respect to some high-level criterion, such that a decision is based on whichever covering value (in the ordered list or a decision tree) is the first to enable a decision to be made (Artinger et al., 2022; Luan et al., 2014). In the case of inference, this ordering is unproblematic—the cues can simply be ordered by how well they predict the relevant criterion. In the case of preference-based choice, however, a consequence of incommensurability is that there is no higher-level covering value to enable a systematic ordering. Thus an assumption of commensurability underpins the choice of which attributes or covering values to consider first, but no commensurability is assumed for the remainder of the choice process. Given our assumption of incommensurability, we must assume that any ordering is produced in some other way.

Compensatory strategies are therefore inapplicable when two or more incommensurable covering values are involved. If choices are made with respect to a single covering value, in contrast, then the decision-maker has a choice: They can use either compensatory or noncompensatory strategies. In this case, computational efficiency and ecological validity will be relevant to the choice of strategy. There is thus a close connection between the distinction between commensurability and incommensurability on the one hand and the distinction between compensatory and noncompensatory strategies on the other. In the face of incommensurability, noncompensatory strategies must be used when different covering values are relevant to the decision. Where there is a single relevant covering value (and hence commensurability), however, either compensatory or noncompensatory strategies can be used.

Our claim of a deep relationship between incommensurability and the use of noncompensatory strategies may seem difficult to reconcile with the existing literature, because evidence for people’s use of lexicographic (noncompensatory) heuristics is typically framed in terms of cues or attributes, not in terms of covering values. However, in the relevant studies, “attributes” and “covering values” typically stand in a one-to-one relation. In weighing up fuel economy versus performance when choosing a car, for example, the relevant covering values (e.g., “saving money,” “enjoying fast driving”) largely relate to different attributes. Thus many experiments on “multiattribute” choice are really experiments on “multi–covering value” choice. (Think, for example, of cameras varying in the attributes of number of pixels and memory capacity or apartments varying in the number of bedrooms and the distance to the nearest bus stop; these attributes all relate to different covering values.) According to the perspective presented here, then, one driving force behind the use of noncompensatory strategies is the incommensurability of the different covering values to which attributes relate, and in such cases, noncompensation is only contingently related to the attributes themselves. If different attributes relate to the same covering value, a compensatory strategy will be possible. But, we suggest, the occasional sense of difficulty in everyday choice typically relates to incommensurable covering values (“Should I choose on the basis of economy or performance?”), not to the relation between different attributes and a single covering value (“To what extent do the car’s weight and engine capacity contribute to its fuel economy?”). Thus, despite the fact that many aspiration-level heuristics may be useful in everyday choice, they cannot help if multiple attributes map onto multiple unique (incommensurable) covering values (Artinger et al., 2022).

Finally, incommensurability helps to explain the close relationship between rank-based strategies for judgment and decision-making (e.g., Ronayne & Brown, 2017; Stewart et al., 2006) and simple heuristics. Decision by sampling (DbS; Stewart et al., 2006) is a rank-based model of how people form subjective valuations (“How satisfied am I with my wages?” “How much do I like this coffee?”). According to DbS, people arrive at these valuations by calculating the relative ranked position of the relevant quantity (e.g., their income) within a comparison sample (e.g., other people’s wages). These estimates of relative rank function as the subjective valuations. The estimates are computed by making a series of simple ordinal comparisons. In valuing one’s income, for example, one might call to mind two lower incomes and six higher incomes, in which case the relative ranked position of one’s own income would be 0.25. DbS was initially developed as an account of people’s subjective valuations of quantities such as incomes, sums of money, amounts of alcohol consumption, and so on (see Brown & Walasek, 2023, for a review). Extensions of DbS to multiattribute choice (Noguchi & Stewart, 2018; Ronayne & Brown, 2017) also involve counting up the number of “wins” achieved by each object in a choice set when compared with other sampled possibilities in multiattribute space.^9^ Furthermore, decision-makers can use the relative ranked position of attribute values within a background distribution to estimate “deal goodness.” For example, a coffee mug at the 80th percentile of the coffee-mug-quality distribution is likely to be perceived as a good deal if its price is at the 20th percentile of the price distribution (Achtypi et al., 2020).

More specifically, noncompensatory decision rules (heuristics) and rank-based models of choice reflect essentially the same response to the problem of incommensurability. A key implication of the idea that covering values are incommensurable is that there is often no advantage to a decision-maker in having better-than-ordinal coding of covering values. This is because ordinal (i.e., rank-based) coding is all that is needed to make choices between objects that differ in the extent to which they satisfy a given covering value. At the same time, better-than-ordinal information could not be used to underpin trade-offs between different covering values. That is, “three units better on economy” could not be traded off against “six units better on performance.” We discuss the implications of rank-based strategies in more detail later but note here that many of the “simple heuristics that make us smart” are themselves types of rank-based strategy.

The foregoing discussion concerns the case of one-to-one mappings between attributes and covering values. Because there is no advantage to be gained by better-than-rank coding of incommensurable covering values, there is equally no gain be had by better-than-rank coding of the associated attribute amounts in such cases. When more than one attribute is relevant to a single covering value, in contrast (as with the example of h-index and conscientiousness both contributing to research value), then better-than-rank representation of attribute amounts could in principle be used to inform decision-making. In such cases, rank-based encoding may still be used, but its use would reflect other reasons (such as coding efficiency; Bhui & Gershman, 2018).

In summary, there is a deep theoretical relationship between, on one hand, incommensurability of different covering values and, on the other hand, people’s widespread use of rank-based and other heuristic strategies for decision-making and choice.

## Arrovian Impossibility

We have noted that if covering values are incommensurable, then there is no advantage in having better-than-ordinal coding of covering values. More specifically, we have argued that, consistent with the wide range of previous literature, rank-based encoding may reflect the incommensurability of value. In this section, we outline the implications of incommensurability-related rank-based coding for some of the most widely studied phenomena in the psychology of decision-making: choice inconsistencies and preference reversals. More specifically, we note that rank-based coding must inevitably lead to the possibility of inconsistency in everyday choice.^10^

The argument exactly parallels the one that Arrow famously made in the context of social welfare theory (Arrow, 1950). Arrow (1950) considered the problem of combining different voters’ preferences over different possible states of the world in order to determine the welfare-maximizing way to organize society. In this case, the incommensurability arises at the level of individuals—it is often (and for present purposes is) assumed that individuals’ well-beings are not commensurable. If so, then as Arrow noted, there is no point in having anything better than a rank ordering of each individual’s preferences over states of the world—because if well-being differences cannot be compared across individuals, a rank ordering of preferences over states of the world is all that can usefully be used to represent individuals’ social preferences. Because of incommensurability, better-than-rank information could not be meaningfully combined across individuals.

Applying this argument to the case of individual decision-making, “attributes” are akin to “voters” and “choice objects” are akin to “possible societies.” For ease of exposition, we first consider the case in which each attribute contributes to one and only one covering value, as in the example about teaching ratings and h-index mapping onto distinct covering values (teaching and research, respectively). In such a case, the problem is of how to meaningfully combine the “votes” that different attributes give to different choice objects. Well-known voting paradoxes are then mirrored in individual decision-making, and this issue has received much attention in prescriptive and normative approaches to the problem of selecting the “best” option from a choice set (Arrow & Raynaud, 1986; Balinski & Laraki, 2010; May, 1954).

Arrow (1950) demonstrated the impossibility of combining individuals’ preference ranks over states of the world into a complete and transitive social ranking while at the same time conforming to a number of plausible criteria. More specifically, no social welfare function can satisfy (all) the conditions that (a) some ordering of preferences must exist (unrestricted domain); (b) ranking of any pair is unaffected by the presence of other options (independence of irrelevant alternatives); (c) if everyone prefers one option over another, then that option should win (Pareto principle); and (c) everyone’s vote has the same weight (nondictatorship) (Maskin & Sen, 2014).

Arrow’s impossibility theorem will also apply at the level of the individual (Arrow & Raynaud, 1986; Balinski & Laraki, 2010; May, 1954). Faced with more than three objects of multiattribute choice, and a preferredness ranking over the objects on each attribute or covering value, Arrow’s theorem limits the extent to which consistent decision-making can result from combining different rank orderings that reflect different attribute or covering-value orderings. We focus on two key (related) issues: intransitivity and preference reversals. Such effects are widely observed in choice (e.g., Kivetz & Simonson, 2000; Tversky, 1969). Our aim is to illustrate why such phenomena must occur under rank-based encoding, even when complete (rank-based) information is available.

We begin with transitivity. Consider an individual facing a choice between three objects, each with three attributes. Assume again that each attribute corresponds to a single covering value.

Table 1 represents the rank ordering of each apartment on each attribute: Apartment 1 has the best location but the highest rent, Apartment 3 is the largest, and so on. How can the decision-maker decide which apartment to choose? A widely studied paradox arises. Apartment 1 seems preferable to Apartment 2 (it is better on two out of three attributes: location and size) and Apartment 2 seems preferable to Apartment 3 (it is better on both location and rent and worse only on size). It might therefore seem that Apartment 1 is the natural choice. But Apartment 3 beats Apartment 1 on a majority of attributes (it ranks more highly on both size and rent). There is therefore no clear choice of apartment based on the ranking of attributes. Intransitivity of this type excludes the possibility of inferring a utility function (e.g., May, 1954). This is the well-known Condorcet paradox, which has been extensively studied in the context of theories of voting and social choice. In those contexts, the objects over which preferences are defined are typically states of the world or election candidates, and the rankings represent the preferences of voters. However, if (as we have argued) different covering values are incommensurable, and hence there is no advantage to better-than-rank coding of such values, the basic theoretical issues remain the same in the case of human decision-making. A decision-making model based on ranks alone will inevitably permit these paradoxes.

**Table 1.** Rankings of Three Apartments on Three Attributes

	Apartment 1	Apartment 2	Apartment 3
Location	1	2	3
Rent	3	1	2
Size	2	3	1

Next, we illustrate how rank-based coding must lead to preference reversals, which are said to occur when Option A is preferred to Option B in one context but B is preferred to A in a different choice context. To illustrate, Table 2 shows the ranked position of three different apartments on seven criteria. Suppose the choice set contains all three apartments. Apartment 1 is clearly the preferred choice; it wins on three attributes while Apartments 2 and 3 each win on only two. Moreover, the mean rank of its attributes (at 13/7 = 1.86) is lower, that is, better, than the mean attribute rank for both Apartment 2 (2.00) and Apartment 3 (2.14). But now consider a restricted choice set, between just Apartments 1 and 3 (i.e., Apartment 2 is no longer available). This case is illustrated in Table 3. The advantage or disadvantage of Apartment 1 versus Apartment 3 remains the same on each attribute as in Table 2 (the larger choice set). But Apartment 3 is now preferred over Apartment 1—it wins on four of the seven attributes. We now have a clear preference reversal and a violation of the principle of “independence of irrelevant alternatives.” Apartment 3 is preferred over Apartment 1 in binary choice, but Apartment 1 is preferred over Apartment 3 when Apartment 2 is added into the choice set.

**Table 2.** Rankings of Three Apartments on Seven Attributes, Such That Apartment 1 > Apartment 2 > Apartment 3

	Apartment 1	Apartment 2	Apartment 3
Location	1	2	3
Rent	3	1	2
Size	2	3	1
Facilities	1	2	3
Decoration	2	3	1
Lighting	1	2	3
Parking	3	1	2
Total	13	14	15

**Table 3.** Rankings of Apartments 1 and 3 From Table 2 After Excluding Apartment 2 From the Choice Set

	Apartment 1	Apartment 3
Location	1	2
Rent	2	1
Size	2	1
Facilities	1	2
Decoration	2	1
Lighting	1	2
Parking	2	1
Total	11	10

In summary, intransitivity and preference reversals in choice are both unavoidable given rank-based coding of covering values. But rank-based coding of covering values reflects the lack of a common scale of value that would make better-than-rank coding worthwhile. Thus preference reversals and intransitivity are an inevitable consequence of incommensurability.

We note that the paradox identified by Arrow in the context of social choice relies on certain assumptions, and hence the equivalent paradox in multiattribute choice relies on analogous assumptions. A key question is, therefore, whether any of these assumptions could be relaxed in models of individual decision-making. There are at least two obvious candidates. First, if decision-makers can use better-than-ordinal labels for covering values, alternative methods of aggregating preferences exist (e.g., see Arrow & Raynaud, 1986; Balinski & Laraki, 2010). Second, as a number of authors have noted, Arrovian paradoxes can be avoided if preference orderings can be represented as single-peaked functions. The conditions under which such functions emerge in multiattribute choice have been specified by Coombs and Avrunin (1977). An important area for future research is whether the solutions that have been proposed to Arrow’s problem in the context of social choice theory can be applied in the psychology of individual choice.

## Counterarguments

In the following section, we address some possible challenges to our claims about the ubiquity and importance of incommensurability.

### Evolutionary fitness

One argument is that evolution has provided us with a single covering value—fitness—with respect to which all choice options can be compared. The suggestion here is not that fitness maximization is a plausible immediate (proximal) cause of decision-making and choice. Perhaps, however, commensurability is assured by the fact that our decision-making apparatus has evolved to serve a single goal, biological fitness?

A full discussion is beyond the scope of the present article. However, we note that evolutionary considerations may also weigh against the idea of a common currency. We have not evolved to maximize some quantity, such as happiness, life satisfaction, or utility. Rather, we have evolved to maximize inclusive fitness—the number of genetically related offspring that we leave behind, weighted according to their degree of relatedness (Hamilton, 1964; McNamara & Houston, 1986). Thus evolutionary theory and economic approaches are similar in that they assume that some quantity (inclusive fitness and utility respectively) is optimized, but fitness considerations do not guarantee that we have evolved to behave as consistent utility maximizers (see Okasha & Binmore, 2012, for extensive discussion). Instead, given the constraints that natural organisms face, we have evolved or can otherwise acquire simple heuristic adaptive preferences (e.g., “prefer not to mate with people you grew up with as children”; Westermarck, 1921). And, as we noted previously, the use of heuristics is an alternative to calculation based on a common currency of value. It is plausible that evolution has endowed us with heuristic preferences both for symmetrical faces and high-calorie drinks (each preference serving an obvious adaptive function) without having endowed us with the ability to trade off such goods against each other (Brown & Walasek, 2023; Hutchinson & Gigerenzer, 2005).^11^

### People can make choices

One possible challenge to our argument is that people do, after all, choose between complex multiattribute objects, both in the lab and in the everyday life. According to Baron (1988), the difficulty of making trade-offs “is a problem in practice, not a problem in principle” (p. 129), and apparent examples of incommensurability are often exaggerated. Baron (1988) denies the importance of incommensurability on the grounds that some choices are extremely easy. Baron illustrates this point with an example of a pregnant person who must decide whether to seek a medical treatment for a dangerous brain infection. The treatment would increase the probability of miscarriage by a very small probability (.00001). The fact that a person would make such a choice without any hesitation suggests, according to Baron, that choice options are indeed commensurable (they can be traded off against one another). A counterargument is that even if people make choices in a seemingly systematic way, such choices are not proof that options are traded off against each other using a common currency (or “common coin” in Baron’s terminology). Multiple noncompensatory decision strategies can produce systematic behavior despite incommensurability of covering values.

It could also be argued that intuitive (as opposed to deliberative) decision-making is immune to the commensurability problem. We suggest that, on the contrary, intuitive decision-making reflects in part a process that makes a reference to only one covering value without considering other relevant and potentially incommensurable values.

### A common currency is found in the brain

For many researchers in the field of neuroeconomics, the correlation between subjective value and brain activation provides convincing evidence for a common currency of value. However, despite the existing evidence, several authors have now presented strong theoretical and empirical arguments against this interpretation (for a recent review, see Hayden & Niv, 2021). As we have noted earlier, many successful models in the judgment and decision-making literature (e.g., many of those based on heuristics) are utility free, and therefore do not require computation of utility by a decision-maker. Yet, many such models may mimic predictions of utility-based models. This point was demonstrated (in the context of interpreting neuroeconomic data) by Piantadosi and Hayden (2015b), who showed that many utility-based binary-choice models can be mimicked by a dimensional prioritization heuristic. This heuristic simply assumes that people calculate the variance of the attributes shared by the choice objects and choose on the basis of the attribute with the highest variance (see also Loomes, 2010). The resulting predictions mimic that of the standard utility model. Correlations between subjective value and brain activation can therefore appear even though no comparison on a single utility-like scale takes place. The difficulty of attributing particular pattern of brain activation to a signal of utility is further exacerbated by the high base rates of activation in the regions typically studied in value-based decision-making (Poldrack, 2011). Similar conclusions can be reached from studies that attempt to map various reinforcement learning strategies onto brain activation. Recent evidence supports models in which decision-makers develop a choice policy or heuristic, rather than tracking expected value of individual choice options (Hayden & Niv, 2021). More generally, evidence for neural representation of a decision-relevant quantity (which could, for example, be the extent to which a particular covering value is satisfied) is not evidence for a higher-level common scale of value.

In acknowledging that utility is a measurable and computable quantity, neuroeconomists make an explicit shift from the “as-if” black box nature of the economic theory toward a process account in which evaluated choice objects are associated with a specific value on a single utility scale. However, this shift brings the issue of incommensurability to the fore. In response to the dimensional prioritization heuristic, for example, Padoa-Schioppa (2015) pointed out that since the heuristic model relies on the computation of variance-of-attribute magnitudes, the model “fails whenever choices are made between qualitatively (incommensurable) goods” (p. 2) and this is problematic because “choices between incommensurable goods are not esoteric cases—they are ubiquitous in the life of humans and other animals” (p. 2). We believe that this argument perfectly reveals the conflict faced by economists who seek to find neural representation of the economic model of choice.

## Related Approaches

Our argument so far builds on the idea that many existing models of choice fail to account for the (here hypothesized) incommensurability of covering values in everyday choice. We have noted that the assumption of commensurability is widespread in models of choice in psychology, economics, and neuroscience. In the following section, we review some theoretical frameworks and concepts that are closely related to the main thesis of this article.

### Reason-based choice

We begin with the fact that heuristic-based models are not the only models of decision-making that are not value based. For example, according to reason-based models (E. J. Johnson et al., 2007; Shafir et al., 1993), decision-makers may struggle to make a choice due to the existence of irreconcilable reasons for picking either option. Reason-based decision-making captures the fact that diverse reasons may reflect different covering values. Because these different covering values are incommensurable, decision-makers must adopt some form of noncompensatory strategy. Indeed, one solution to the conflict is to adopt a single-rule decision strategy (e.g., choose experience rather than material possessions; do not buy meat), which is akin to prioritizing a single covering value (Levi, 1986). In a similar vein, a decision-maker may assume a pseudo-rational strategy, focusing only on financial aspects (lay economism), choosing on the basis of pure performance (lay scientism), or prioritizing current goals, like satisfying hunger or need for comfort (lay functionalism) (Hsee & Hastie, 2006).

### Goal-based decision-making

Goal-based models of choice typically seek to explain departures from utility- or value-based models in terms of the activation of different, and often competing, goals and motivations (Carlson et al., 2008; van Osselaer et al., 2005; van Osselaer & Janiszewski, 2012). Describing each model is beyond the scope of the present article, but we note here that many existing goal-based theories typically differ with respect to the source of goal activation (e.g., choice context, goal priming) and classification of goal types (e.g., psychological needs, hedonic, social, physiological). Goal activation can determine attribute weighting, and this is commonly referred to as goal-based evaluation (Fishbach & Dhar, 2007).

Goal-based models differ from our own account in their assumption about what happens when more than one goal is active at the time of choice (see Type IV cases as defined earlier). Some models of goal-based decision-making do not explain how multiple attribute weights based on separate goals are integrated. Others state that goals can be traded off against one another, often relying on affect as a common currency (e.g., Brendl et al., 2002; Carlson et al., 2008; Fishbach & Dhar, 2007; Krantz & Kunreuther, 2007; van Osselaer & Janiszewski, 2012). In the presence of goal conflict, some trade-off resolutions involve satisfaction of multiple goals, such as when individuals switch between goals over time (goal balancing) or use past actions as an “excuse” for the current behavior (goal licensing) (Mullen & Monin, 2016; Shaddy et al., 2021).

Higher-order goals have also been included in JDM models of choice. For example, in the influential adaptive decision-maker framework (Payne et al., 1993), individuals making choices are argued to trade off goals of (a) minimizing cognitive effort, (b) maximizing decision accuracy, (c) reducing negative affect, and (d) maximizing ease of justification (note that [a] and [b] are the primary features of the adaptive decision-maker in Payne et al., 1993).

In many applied settings, the presence of conflicting goals and values lies at the heart of tools and methodologies in decision analysis. Broadly, this approach is designed to help decision-makers make complex decisions using a common (utility-based) currency. The very popularity of such tool kits in business and management exemplifies the pervasiveness of the common-currency assumption.

In the JDM literature, some have noted that choices may not reveal true preferences due to personally held goals of an individual (Baron, 2004). Yet, the importance of goals in models of JDM has been largely ignored, most likely because they make the task of modeling choice behavior computationally intractable (Arkes et al., 2016). More recently, Bergner et al. (2019) proposed a modeling architecture (Voting Agent Model of Preferences – VAMP model) in which decision-makers generate multiple preference profiles based on their personal goals. The authors show how different methods of aggregating rank orderings of choice options may produce context effects. More specifically, scoring rules that fall between the plurality vote (where all points go to the most preferred option) and Borda count procedure (where each option receives *n* – *x* points, where *x* represents options’ relative rank) can give rise to the three classical context effects: attraction, compromise, and similarity effects. The VAMP model therefore captures some of the proposals in the present article, namely, that multiple covering values (or goals) may correspond to distinct weighting of attributes and that aggregations of covering values may produce inconsistencies in choice. At the same time, however, preference orderings in the VAMP model are based on random utility, thus assuming an underlying common currency of value.

### Empirical tests of incommensurability

The view presented so far is that incommensurability rarely features in the debates about psychologically motivated models of JDM. Although the same can be said about empirical studies of incommensurability, a few notable exceptions exist. For example, in experimental studies conducted by Deparis et al. (2012, 2015; see also Beattie & Barlas, 2001), participants were asked to choose between apartments that varied in rent and distance from the city center. In addition to being able to express preference between any two options, participants in some conditions could also state that they are indifferent or that they “cannot compare” available alternatives. The “cannot compare” response was chosen on almost 20% of occasions, suggesting that participants had little trouble in distinguishing indifference from incommensurability. Furthermore, when participants indicated that they could not make a comparison, they did so when they otherwise would have said that they are indifferent. This suggests that people may state that they are indifferent when they in fact find it difficult or impossible to decide.

We acknowledge that the incommensurability assumption makes it even harder to study everyday decision-making. How can we experimentally elicit covering values and demonstrate how decision-makers overcome their incommensurability? One recent but promising methodological approach involves a combination of cognitive modeling, reason-based choice, and natural language processing. By quantifying and modeling reasons using language data, researchers have been able to demonstrate latent attributes (or covering values) underpinning people’s decisions about risks, consumer goods, ethics, or food (Gandhi et al., 2022; Zhao et al., 2021). Similar methods could be used to understand failures to make a choice, such as in situations where a decision-maker is indifferent, states that options cannot be compared, or opts to defer a decision.

## Summary and Conclusion

A primary goal of this article is to encourage discussion of value incommensurability by behavioral and decision scientists. We have argued that that the assumption of a single scale of value is ubiquitous in economics, psychology, and neuroscience and presented an alternative view in which incommensurability prevents people from choosing objects on the basis of a single quantity, such as utility, fitness, or well-being. We have shown that some lexicographic strategies address the question of incommensurability and may provide a method for choosing when multiple covering values are present. We also have noted that if covering values are incommensurable, a decision-maker often has no use for anything more than rank information about covering values. This reliance on rank, in turn, places constraints on how consistent decisions can be made (based on Arrow’s impossibility theorem). Although the problem of choosing the “best” option when options are ranked according to multiple criteria or different people has received much attention in diverse domains, including voting theory (Balinski & Laraki, 2010), engineering (Franceschini et al., 2022), computer science (Bisdorff, 2022), operations research (Arrow & Raynaud, 1986), and group decision-making (Hastie & Kameda, 2005), it is not prominent in psychological models of individual choice in relation to the incommensurability of value. Some rank-based heuristics for judgment and decision-making can, however, we have suggested, be seen as a response to the incommensurability of value.

Our message can be seen as a positive one in that many apparent paradoxes and inconsistencies in decision-making appear less problematic when no longer viewed through the lens of assumed value commensurability. At the same time, value incommensurability places clear limits on the extent to which choices can be justified as well as the extent to which they can be guaranteed to be consistent (Sperber & Mercier, 2018). Value incommensurability therefore has implications for wider issues, such as what counts as evidence for “irrationality.” In the context of simple choice, irrationality is often thought of in terms of “inconsistency” (as evidenced by, for example, preference reversals). However, if there is no single universal scale of value, preference inconsistency can be seen as inconsistent only if it occurs with respect to a particular covering value. To put this another way, if preferences are incomplete in a way that does not reflect insufficient learning or reflection, preference reversals need not be seen as inconsistent or indeed “irrational” if they reflect choices that have been made with respect to different covering values (see Levi, 1986, for an extensive and valuable discussion).^12^ Future models of decision-making will, we suggest, need to confront the issue of incommensurability directly, both theoretically and empirically. At least, if no single value to be maximized exists, then current models will be unable to explain choices except when a single covering value is explicit and/or the choice scenario does not require consideration of additional covering values.

Finally, our argument relates to wider issues concerning the existence or nonexistence of internal mental scales for measurement and the representation of magnitudes and values. The existence of multiple incommensurable values is widely assumed in fields such as phenomenology, political science, and philosophy. It also accords with the everyday intuition that not everything can be reduced to monetary values. Thus the assumption of a common scale of value, while ubiquitous in models of choice within both economics and JDM research, is perhaps best seen as the exception rather than the rule. Even within psychology, however, the idea of universal psychological measurement scales—of the type that might be assumed to underpin value representation—is often assumed to be defunct. First, people’s subjective judgments of quantities such as the weight of an object or the loudness of a sound are systematically context sensitive in ways not predicted by traditional psychophysical laws, such as Fechner’s law or Stevens’s law (see Parducci, 1982; Stewart et al., 2005). Second, people’s subjective judgments of simple magnitudes are, as a matter of empirical fact, not commensurable. Thus people cannot, for example, match a given loudness to a specific brightness or length in any context-independent way (Laming, 1997). Such findings provided evidence against the “new psychophysics” (Stevens, 1960) approach, according to which subjective magnitudes of stimuli from different sensory modalities can be represented on a common scale (Stevens, 1970). Although subjective valuations of different attributes could in principle be commensurable even if subjective magnitude judgements are not, our claims about the incommensurability of values appear consistent with psychological evidence for incommensurability more generally.

## Transparency

*Action Editor:* Mirta Galesic

*Editor:* Interim Editorial Panel

## Notes

1. See https://en.wikipedia.org/wiki/Apples_and_oranges.

2. Marx does not use the language of commensurability and comparability directly but does speak at length of the different qualities (and use values) of different commodities.

3. We note that some models of choice within a post-Keynesian framework deny the idea of commensurability across different levels of a hierarchy of needs (see Lavoie, 2014).

4. See (Levi, 1986) and (Elster, 2015) for different varieties of “comparability.”

5. Our proposal concerning incommensurability of covering values is also distinct from the concepts of comparability, dimensional commensurability, and evaluability that have been used in describing multiattribute consumer choice (M. D. Johnson, 1984). Comparability has been defined as the degree of overlap between attributes of two choice objects (e.g., the attribute overlap between two cameras is greater than the overlap between a camera and a fridge; M. D. Johnson, 1986; Markman & Medin, 1995). Dimensional commensurability, on the other hand, refers to cases where, in the presence of perfect comparability (as defined here), some attributes are missing (e.g., when comparing two cameras, information about the memory size of one is not available; Slovic & MacPhillamy, 1974). Finally, the notion of evaluability refers to the relation between subjective evaluations and attribute scores (Hsee & Zhang, 2010)—for example, how decision-makers choose their willingness to pay for diamonds of different quality (number of carats). One relevant and robust finding on evaluability is that preference reversals can occur when options are evaluated individually versus when they are evaluated jointly. On a separate note, one could think about covering values as context-dependent utilities or goals. Although both concepts have been used in economics and psychology, neither is directly used to address the issue of incommensurability. We return to the discussion of goals in the Related Approaches section.

6. We note that “liking” and “wanting,” like experienced utility and decision utility, are different constructs (see, e.g., Berridge, 1996). This distinction, while important, is orthogonal to the distinction between commensurable and incommensurable values: Wanting and liking might involve either the same or different covering values.

7. Different choice contexts may of course induce reliance on different covering values; the point is that the differing attribute weights are being produced not directly by the attributes of other options but only indirectly.

8. Such changes are typically averaged over in economic models of choice (for example, see Sen, 1977), but are illustrative in the present context.

9. If two attributes are price and quality, for example, each option gains a “point” for each comparison-sample item that it dominates (i.e., each item that it is better than on both the price and quality dimensions).

10. We do not mean to imply that such inconsistency is necessarily problematic in practice (Arkes et al., 2016) and note also arguments that phenomena such as preference reversals may under some circumstances be adaptive (McNamara et al., 2014).

11. Although there are cases where very different attributes (e.g., the energy value of a food and the distance to its source) may be traded off using a common currency (the calories gained by consumption and lost by traveling), we assume that such cases are rare in everyday multiattribute choice.

12. Compare also Elster’s (2015) idea of “non-irrationality.”
